# Availability of essential medicines during the COVID-19 pandemic: A qualitative study examining experiences and level of preparedness in Kenya

**DOI:** 10.1371/journal.pgph.0002547

**Published:** 2024-07-29

**Authors:** Joseph Odhiambo Onyango, Dosila Ogira, Gilbert Kokwaro

**Affiliations:** Institute of Healthcare Management, Strathmore Business School, Strathmore University, Nairobi, Kenya; UIndian Council of Medical Research, INDIA

## Abstract

This study examines the impact of the COVID-19 pandemic on the availability of essential medicines in Kenya and suggests actionable measures to enhance the country’s preparedness for future pandemics. Utilising a cross-sectional qualitative design, the research combines a systematic review of the literature and 20 key informant interviews to provide a comprehensive analysis. The initial response to the pandemic involved resource reallocation, disrupting the procurement of essential medicines at national and county levels. Inefficiencies in these systems resulted in shortages and wastages of crucial medicines, exposing vulnerabilities in the health system. Furthermore, the study reveals regulatory policy weaknesses in Kenya, such as an inadequate legal framework for domestic pharmaceutical manufacturing and conflicting policies hindering medicine availability. The study recommends a multifaceted policy approach to ensure essential medicine availability during crises. Key recommendations include strengthening financial systems through increased government investments and innovative funding mechanisms, implementing price regulation policies, and enhancing the resilience of supply chain and procurement systems. Collaboration among various supply systems is essential to prevent stock-outs. Strengthening legislation and regulatory policies, increasing domestic pharmaceutical manufacturing capacity, and investing in health information systems are vital for sustained self-sufficiency and efficient service delivery. These comprehensive measures are essential to promote essential medicine availability, safeguard public health, and enhance resilience during crises.

## Introduction

On January 23, 2020, the World Health Organization’s (WHO) International Health Regulations (IHR) Emergency Committee, in response to the new coronavirus disease 2019 (COVID-19) first detected in Wuhan China, convened and issued a directive that: "all countries should be prepared for containment, including active surveillance, early detection, isolation and case management, contact tracing and prevention of onward spread of 2019-nCoV infection, and to share full data with WHO” [1]. Based on recommendations from the Committee, the virus was declared a public health emergency of international concern on 30^th^ January 2020 [2].

One of the areas that was primarily affected by the pandemic is the availability of essential medicines. Previous studies on epidemics and pandemics have reported an increased rate of infections due to disruption of medical supplies and products such as during the 2014 West Africa Ebola outbreak [3]. Similarly, the coronavirus was initially seen to affect supplies in both high- and low-income countries negatively. For instance, scholarly investigations undertaken to ascertain the impact of COVID-19 in high resource settings such as the United States and Saudi Arabia revealed shortages of ten or more essential medicines at the onset of the pandemic [4–7]. Chloroquine and hydroxychloroquine were some of the medicines enlisted since they were believed to alleviate the COVID-19 symptoms and reduce its severity [8]. Additionally, shortages of essential medicines were also attributed to the disruption in the global pharmaceutical supply chain [9].

Most Low and Middle-Income Countries (LMICs) experienced a greater risk of medical product shortages due to their already weak health systems and low resource setting than High-Income Countries (HICs) [10]. These shortages were further exacerbated by low production capacity and heavy reliance on developed countries for raw materials and finished products [11]. For instance, countries like China and India who have long manufactured and exported active pharmaceutical ingredients to LMICs had reduced their production capacities [12]. Additionally, mitigation measures to control infections globally resulted in lockdowns and limited movement of people and products, affecting the supply of essential medicines [13].

Currently, the Kenyan health system grapples with conflicting demands arising from both communicable and non-communicable diseases, exacerbated by the highly fragmented nature of its pharmaceutical sector [14, 15]. Additionally, the Country still heavily relies on pharmaceuticals from other countries with approximately 70% being sourced from India (37%), Europe (20%), China (9%), the United States (6%), and South Africa (4%). In comparison, the remaining 28% is domestically manufactured [15, 16]. The COVID-19 pandemic saw most countries, including HICs, focussing on their needs hence LMICs such as Kenya were forced to look for alternative ways of meeting their needs [17]. To depict this, early findings in Kenya indicated shortages in essential medicine including the Internationally Controlled Essential Medicines (ICEMs) [18]. However, an innovative supply-chain strategy was crafted in rural western Kenya to ensure the continual access and provision of essential medicines to patients amidst the pandemic. This involved establishing a revolving fund pharmacy model, strategically stocking crucial medicines in constituent health facilities for easy patient access. The success of this model relied on leveraging the longstanding partnership between Academic Model Providing Access to Healthcare (AMPATH) and the local government in western Kenya [19].

Universal Health Coverage (UHC) is the primary agenda in Kenya, and it aims to provide accessible and affordable healthcare to the population. However, pursuing this objective faced significant challenges due to the impact of the COVID-19 pandemic [20]. For instance, KES 11.3 billion was reallocated from the UHC reservoir to boost the COVID-19 kitty at the beginning of the pandemic risking other aspects such as availing essential drugs [21, 22]. Therefore, to ensure the availability of essential medicines and achieve UHC amidst other competing healthcare needs, there is need for innovative solutions to match especially during unprecedented times [23]. This study sought to understand how the COVID-19 pandemic has impacted the access to essential drugs in Kenya and some of the actions stakeholders can take to improve the country’s preparedness for future pandemics. Maintaining continued health progress will depend on how the health policymakers navigate this pandemic through emergency preparedness. The main aim of this study was to understand how the COVID-19 pandemic has affected the availability of essential medicines in Kenya. Specifically, we (a) Examined the health system gaps impacting the access and provision of essential medicines and (b) Identified opportunities to enhance the country’s preparedness for future pandemics.

## Methods

### Ethics statement

This study obtained ethical approval from Strathmore University’s Institutional Review Board (Reference number: SU-IERC1050/21) and the National Commission for Science, Technology and Innovation (License no: NACOSTI/P/21/12474). The study was conducted virtually due to the social distancing measures from COVID-19, and all participants were requested to consent verbally. A study information sheet contains information on 1) the title of the Study and why it is being carried out, 2) which participants were going to be involved and why, 3) time demands for the participants, 4) the voluntary nature of participation into the Study, 5) assurance of confidentiality once they agree to participate and 6) how the data provided will be utilised; was shared with participants on email a few days before the interview was set. All recordings and transcripts were deidentified to protect the interviewee’s confidentiality.

### Study design and sampling technique

This study employed a cross-sectional qualitative design to meet its objectives [24]. We chose qualitative methods to enable a deeper understanding of the complex interplay between health system gaps and the accessibility of crucial essential medicines. A qualitative approach is fitting for understanding the complexities of challenges and improvement opportunities in ensuring the availability of crucial medicines during a pandemic. It offers a holistic view, providing rich insights that quantitative methods may not fully capture [25]. Additionally, a cross-sectional approach allows for data collection at a single point in time, offering a comprehensive view of the prevailing health system gaps and opportunities for future pandemic preparedness [26].

Additionally, due to its qualitative nature, the participants were selected purposively, focusing on those with experience in the sector who were willing to share the information. Interviewees were asked about their knowledge and experience with the availability of essential medicines during COVID-19. In this study, after the 20th interview, no new insights were gained, and thematic redundancy was observed, indicating data saturation. This study was conducted during the surge of COVID-19 from July 2021 and was completed in July 2022.

### Data acquisition

We integrated literature review and key informant interviews to collect data and achieve the study objectives. For the literature review, a comprehensive review of grey and peer-reviewed literature on the impact of COVID-19 on essential medicines was carried out sequentially from the global, regional, and local contexts to understand better and explain the phenomenon. For the key informant interviews, we carried out 20 semi-structured interviews. The predefined questions provided a framework for consistency, ensuring that key topics were covered while also allowing participants the flexibility to express their perspectives in depth. The study’s focus on health system gaps and opportunities during a pandemic necessitated individualized insights. Semi-structured interviews allowed for in-depth exploration of each participant’s experiences and perceptions, facilitating a comprehensive understanding.

Additionally, individual interviews created a comfortable space for participants to openly discuss sensitive healthcare and policy issues without the influence of group dynamics, particularly relevant when addressing pandemic-related impacts on healthcare access [27]. The key informants included representatives from (i) National government-2, (ii) Sub-national County government-7, (iii) Health professional bodies-2, (iv) Pharmaceutical manufacturers-5, (v) NGOs and CSO representatives-4. Due to the initial movement restrictions resulting from the COVID-19 pandemic, the interviews were carried out virtually. All interviews were conducted via Zoom videoconferencing in English, averaging one hour in duration. Interviews were recorded only after oral consent was obtained from the participants.

### Data analysis

A professional transcription service conducted all the audio file transcription. One team member (DO), ensured transcription accuracy by reviewing all transcripts, comparing them to the audio files, and making necessary edits. The team (DO, JO, GK) began by reading through all the transcripts which had been transcribed verbatim. After the data familiarization, an initial codebook was developed by one researcher (DO) drawing on the study objectives and integrating emerging themes from the interviews. This codebook was then validated by the research team members (JO, GK) and through regular meetings to discuss and refine codes, ensuring consistency in the coding process. After refining the codebook, the interviews were coded by (DO) using NVIVO software. The findings were analysed by (DO) and presented based on the prevalent views, which were then reviewed by the other study team members (JO, GK).

Triangulation and response validation strategies were implemented to enhance the robustness and credibility of the study findings. Multiple data sources, such as interviews with various stakeholders, were employed to understand the research topic comprehensively. This methodological triangulation involved cross-verifying information from different sources to strengthen the study’s validity. Furthermore, member checking, a form of response validation, was conducted. Preliminary findings were shared with participants, allowing them to review and confirm the accuracy of the interpretations.

### Findings

The findings are categorized into six broad themes outlining the experiences, challenges, and opportunities about the availability of essential medicines during the COVID-19 pandemic in Kenya. These include (i) healthcare financing of essential medicines, (ii) supply chain and procurement system for essential medicines, (iii) regulatory policies and manufacturing capacity of essential medicines, (iv) human resource for health training on production of essential medicines, (v) health information system on essential medicines, and (vi) the role of the public versus private sector in accessing essential medicines and promoting UHC.

### Healthcare financing for essential medicines

Most of the respondents reported that the initial reaction by the Country involved the revision of priority gaps which resulted in the diversion of resources, including financial resources towards management of the COVID-19 pandemic. For instance, it was mentioned that the price of some non-pharmaceutical commodities used for containment, such as surgical gloves and masks went up almost 20 times, and more funds had to be allocated towards their purchase. This priority shift was seen to affect other aspects in the healthcare sector including the procurement of essential medicines both at the National and County levels.

*“So, pre-corona we could supplement well what KEMSA didn’t have. But Corona came in and things went upside down. All the County funds that were meant for essential commodities were devised to buy preventive commodities for coronavirus and it meant that now we had less commodities that were always available with us. So, it meant fill rate prescriptions and essential pharmaceutical availability went down.”*

***Key Informant 7***

Some respondents faulted the Country’s emergency preparation due to lack of financial reserves dedicated towards emergency response. This was seen to slow down organic and coordinated response including acquisition of essential medicines. However, as the pandemic progressed, some counties called for emergency meetings to reallocate funds for purchasing of some of the essential medicine that were running out of stock then. Additionally, high level of donor dependency for some essential medicines was also outlined as a challenge and if there was a reduction, this was seen to affect the flow of essential medicines.

Additional recommendations to be adopted to alleviate challenges facing financing of essential medicines in the event of a future pandemic were made by respondents. First, some respondents expressed a need for improving financial policies through establishing facility improvement funds and financial reservoirs that could be retained at the facility level. This was seen as a sustainable way of ensuring that commodities are available and can act as a buffer in the event of a pandemic and increase the accessibility of medicines to patients. Secondly, suggestions were made to improve financial forecast to matchup with the financial allocation of essential medicines especially in the public sector in the Country. This was seen as ensuring access to essential medicines hence promoting Universal Healthcare Coverage (UHC).

*“So, we should start building up some kind of funding for any eventuality or calamity that might be used for essential medicines—we don’t have that kind of funds sitting for free for such kind of scenarios.”*

***Key Informant 11***

### Supply chain and procurement systems for essential medicines

During the COVID-19 pandemic the supply chain system was heavily disrupted globally. Respondents mentioned that the rigidity of movement attributed by measures such as lockdown slowed down the production and access to the essential medicines. For instance, manufacturing countries like India and China that produce active pharmaceutical ingredients and supply Kenya had highly reduced their exportation rate, with some factories shutting down because of COVID-19 cases, negatively impacting production of essential medicines. Additionally, with reduction in production, there was also an increase in competition for raw materials and a subsequent increase in cost of essential medicines across countries due to high demand and low supply.

Almost all respondents from sub-national level reported that there was deviation from what was a routinely smooth supply chain and procurement system cascading from the National government with extended lead time. With many efforts being redirected towards the containment of the pandemic, it was mentioned that shortages were experienced even for some of the key essential medicines such as paracetamol. However, some respondents noted that not all essential medicines were affected in the same way, and some of the shortages were artificial due to consumers’ overreliance on brand prescription and the preceding prescribing pattern.

*“Some patients only believe in the products that’s written on their prescription and they were not willing to switch maybe to another brand or to a generic …that’s what I mean by artificial shortage, some patients on blood pressure medication would feel that the products they’ve been on for so long is not there and it’s difficult to switch them to a similar product, but another brand or a generic.”*

***Key Informant 15***

However, as the pandemic progressed, the government took swift measures to alleviate the hurdles faced by the supply chain. For instance, in terms of limited transportation of essential medicines due to lockdown, the government issued permits to distributors considered essential workers. Additionally, other counties leveraged previous good working relationships with local private suppliers, which helped them acquire critical essential medicines at the time. Other innovative ways of strengthening the supply chain were also witnessed; for instance, some counties restructured their systems to distribute essential medicines to smaller facilities. This was seen to help patients access essential drugs within their locality without travelling longer distances.

***“Then also we are coming up with a pilot which we are still in our initial stages of*** *implementation, this patient who used to travel to the bigger health facilities to attend their clinics and get their drugs refilled…we are sending drugs to these smaller facilities and also accompanied with health workers who have experience with dealing with the noncommunicable diseases so that this patient doesn’t have to travel long distance to go to the bigger facilities.”*

***Key Informant 19***

Recommendations were also made to ensure that there is always a focus on the supply and access of essential medicines. For instance, it was mentioned that the supply chain systems should be robust by setting up real-time systems, providing a good quality management system process, and encouraging stockpiling to ensure that the supply and access of essential medicines are maintained.

### Regulatory policies and manufacturing capacity of essential medicines

Almost all the respondents faulted the government for having rigid regulatory policies and systems that were not responsive to unique situations such as the pandemic. Despite the emergency, the Country took time to introduce alternatives for outsourcing essential medicines or giving approvals, which would have improved the response rate and interventions at the time. Additionally, there were initial cases of corruption witnessed at the Country’s main medical supplier to counties, which negatively affected the stock levels and flow of essential medicines.

*“And also, you know, for us we work within the county so our main supplier is the Kenya Medical Supplies Agency, KEMSA, and with whatever happened, the gaps in accountability, it messed up the stock levels and as a result, those of us who depend on that particular body as the main supplier were directly affected. And as I said overall there was that decrease in supply.”*

***Key Informant 6***

It was also noted that the government policies have not been coherent or conclusive of promoting local manufacturing of essential medicines despite the Country’s ability and capacity. Some respondents pointed out gaps, including unfavorable tax brackets for the local manufacturers leading to high manufacturing costs. As a result, respondents felt that this created unfair competition for the local manufacturers. Additionally, the Country was seen to heavily rely on importation of active pharmaceutical ingredients to manufacture finished products, contributing to the shortage of essential medicines during the pandemic.

Amidst the challenges, some respondents noted that there was progressive response from the government which saw introduction of regulatory approaches aimed at strengthening and supporting local production of essential medicines.

*“Right now, if you go to the national treasury there is a finance bill speaking to aspects of exemptions and tax reliefs to local manufacturers to be able to start manufacturing in the county.”*

***Key Informant 12***

Some respondents felt that improving local manufacturing capacity for the Country to be more self-reliant would have sealed the gap and improved the shortage of essential medicines. Expanding the manufacturing portfolio even to include active pharmaceutical ingredients that produce various finished products was seen as one of the ways.

*“If you look at the essential list, you’ll find a lot of the big manufacturing sites here in Kenya are manufacturing only three or four elements, mainly the analgesic portfolio, where you have your paracetamol, and a few of the anti-infective ’s where you have your antibiotics being produced. So, there is need to scale up local production and local manufacturing to a higher level whereby we reduce that reliance to external manufacturing.”*

***Key Informant 2***

Other recommendations included strengthening the regulatory authority to encourage foreign investors to venture in production in the Country and incentivizing local manufacturers through improving tax policies to reduce cost of production. Strengthening multisectoral and cross-country ties was also highlighted to improve the availability of essential medicines.

### Human resource for health training on the production of essential medicines

Few of the respondents pointed out a gap in the country’s curriculum and training, especially around the production of essential medicines. They felt that the current curriculum had limited content on the production of complicated molecules, and there was a need to expand it and spur more manufacturing, even into human vaccines, some of which are on the essential medicines list.

*“The other area is the area of training, and this is another area that is missing especially looking at the impact of the regulatory science in this and industrial pharmacy. The career of the pharmacists needs really to be tailored so that it is in line to with these requirements.”*

***Key Informant 4***

However, one of the respondents mentioned that the government had initiated strategies to incorporate these aspects of training. They illustrate;

***“And for XXX, yes, we are starting. And in fact, we’ve started as an in-house, we’ve developed*** *courses for training of our recruits that come in…we’ve developed it as an online system so; eventually, we are hopeful that we shall be able to stimulate the expansion of the regulatory science into universities because it will also really help.”*

***Key Informant 14***

Recommendations were made on building the capacity of healthcare professionals through targeted training programs on production of essential medicine. This was seen as a sustainable way of addressing the needs of the Country in the event of a future pandemic.

### Health information system on essential medicines

It was noted that existing information system was not robust and there were instances of downtime which affected the ordering of essential medicines. Additionally, process documentation for future forecasting and quality management system was also seen as a point of weakness both at the national and County levels.

*“In terms of the procurement and information system, as I mentioned, there had been a slowdown, based on the lack of the systems being independently run. There is a lot of dependence on individuals rather than the system, so if that individual is unwell then the process stalls.”*

***Key Informant 13***

However, few respondents observed that the pandemic created opportunities to improve the health information system concerning availability of essential medicines. For instance, the pandemic heightened the need to improve and strengthen supply chains through digitization. Additionally, in some instances there was a move from manual to automated database systems that can be used to forecast the use and need of essential medicines in the Country.

*“There was a lot of improvement towards supply chain and even digitalization… you heard of a system called ‘****chanjo’*** *(immunization), it was actually conceived to manage vaccines and the supply chain.”*

***Key Informant 12***

Recommendations were made to set up solid, secure systems with a clear quality management system protocol, ensuring that despite situations of calamities or pandemics, the systems continue to run. Additionally, respondents reported a need for better data collection and documentation improving data collection that can be used for fact-based decision making.

### The role of the public versus private sector in accessing essential medicines and promoting Universal Health Coverage (UHC)

The respondents highlighted the long-standing gaps in the availability of essential medicines in the Country’s public health system, which were alleviated by the COVID-19 pandemic. Some of them attributed this to the inefficiencies in supply chain forecasting in the public sector, which in some instances has resulted in expiries and stockouts of essential medicines. They believed that harmonization of the public and private supply chains could reduce the mismatch and improve the availability of essential medicines.

*“In terms of downstream manufacturing, I would say in the private sector that is sorted…that usually flows because it market-driven and so it’s quite efficient. But in the public sector we don’t see that type of efficiency and that’s why we see expiries, stock-outs and poor storage at KEMSA. So, for the downstream public sector, we have to strengthen KEMSA because now counties must buy from them by law*.

***Key Informant 14***

Some of the respondents, most of whom were from the public sector, highlighted the opportunities for collaboration with the private sector to ensure continuity of the flow of essential medicines. They noted that the private sector tried to bridge some of the gaps that the government could not sort out during the COVID-19 pandemic.

*“Yes, to some extent we still have some of our stakeholders being private entities, mainly those supplying us with medicines through the tender system. They came in handy because when we were low on finances, we could negotiate with them and they supplied us on a debt.”*

***Key Informant 7***

There was recognition and consensus among respondents that the private sector played a very important role in terms of promoting availability of essential medicines. Recommendations were brought forth to foster collaboration between the public and private sector as it was seen as the most sustainable way of achieving UHC even during unprecedented times.

*“You know half of Kenyans go to the private sector for their healthcare. So, we have to include private sector in UHC and all levels of private sector; the private hospitals, the private labs, the private pharmacies. So, we cannot force UHC models on people. It has to fit into the behavior of the clients of healthcare*.”

***Key Informant 1***

## Discussions

In this discussion section, we analyze the findings of our study, which aimed to understand how the COVID-19 pandemic has affected the availability of essential medicines in Kenya. We focus on the challenges faced by the healthcare system during the global health crisis, shedding light on immediate impacts and proposing strategies for future resilience. By exploring these dynamics, we contribute insights to the ongoing discussion on strengthening healthcare systems in the face of global health emergencies, specifically regarding essential medicines.

The COVID-19 pandemic underscored the significance of setting up both local and international procurement and supply chain systems that are well-coordinated and adaptable to shocks that health systems can experience as highlighted by this study. Inefficiencies in supply chains resulted in shortages and even wastages of essential medicines. Although other private and faith-based suppliers of essential medicines exist in Kenya, the bulk of the procurement is done by KEMSA, which majorly serves public institutions and the population (the majority of who are poor) [15, 28]. The Country witnessed corruption and lack of transparency at KEMSA which marred the procurement, distribution and use of essential and non-essential medicines and products during the COVID-19 pandemic [29, 30]. To prevent potential inefficiencies in the future, the Country could mitigate restrictions imposed on public sector facilities. Rather than exclusively relying on KEMSA for procurement, a diversified approach incorporating public, private, and faith-based/NGO supply systems can be adopted. This diversified strategy would be complementary, ensuring the procurement and distribution of essential medicines, even in times of crisis. Literature denotes that harmonization and collaboration by these sectors can reduce stock-outs and optimize availability of essential medicines through ways such as through pooled procurement and innovative supply chain models [31–34].

Pharmaceutical laws and regulations, create a basis under which operations such as manufacturing, trade and use of essential medicines are safeguarded. Whereas weak and conflicting policies can result in inefficiencies, responsive policies can help promote standards that will ensure availability of essential medicines [35–37]. Findings from our study showed that the pandemic exposed some of the weaknesses of regulatory policies in the Country such as; inadequate legal framework to support domestic pharmaceutical manufacturing capabilities, conflicting policies and over-regulation of the pharmaceutical sector that hindered availability and accessibility of essential medicines. While the country has undertaken concessional efforts, such as signing the African Medicines Agency treaty and being a member state of the East African Community-Medicines Regulatory Harmonization (EAC-MRH), there remains a critical need to amplify investments in the production and manufacturing portfolio of essential medicines and products [38–41]. As the literature denotes, crucial to this will be investing in the Country’s technology, infrastructure and human resource capacity, which can promote local production [40, 42, 43]. Additionally, incentivizing local production, such as through the establishment of a conducive legislative framework, increases local production [37, 44]. These legislations can include price controls, tax relief for manufacturers and the overall establishment of good manufacturing practices (GMP) that attract local and international investments in production [41, 42, 44].

Various limitations of this study are acknowledged. First, the study relied on key informant interviews with multiple stakeholders within the healthcare system, potentially introducing a bias based on the perspectives and experiences of the interviewees. Although we mitigated this with document review, future research could incorporate a more diverse and comprehensive sampling strategy, ensuring representation from a wide range of stakeholders, including patients. Furthermore, while the study endeavours to identify opportunities for enhancing pandemic preparedness, the dynamic nature of the healthcare landscape suggests that the identified opportunities may evolve over time. Despite these limitations, our study provides valuable insights into the complex interplay between health system dynamics and the availability of essential medicines during a pandemic, serving as a foundation for further research and policy considerations.

## Conclusion

In conclusion, this study recommends a multifaceted policy approach to ensure the availability of essential medicines, particularly during crises like the COVID-19 pandemic. Key recommendations include strengthening financial systems for essential medicines through increased government investments and innovative funding mechanisms, implementing price regulation policies to enhance accessibility, and bolstering the resilience of supply chain and procurement systems. Collaboration between various supply systems is crucial to prevent stock-outs. Furthermore, strengthening legislation and regulatory policies, increasing domestic pharmaceutical manufacturing capacity, and investing in health information systems, including digitisation, are vital for sustained self-sufficiency and efficient service delivery. Implementing these comprehensive policy measures is essential to promote the availability of essential medicines, safeguard public health, and enhance resilience in times of crisis.
